# Phosphorous-Nitrogen Modification of Epoxy Grafted Poly-Acrylic Resin: Synergistic Flame Retardment Effect

**DOI:** 10.3390/polym13162826

**Published:** 2021-08-23

**Authors:** Chao Liu, Hui Qiao, Guilong Xu, Yun Liang, Jin Yang, Jian Hu

**Affiliations:** 1 National Engineering Research Center of Papermaking and Pollution Control, South China University of Technology, Guangzhou 510640, China; amt242523@163.com (C.L.); qiaohui1005@gmail.com (H.Q.); liangyun@scut.edu.cn (Y.L.); yangjin@scut.edu.cn (J.Y.); ppjhu@scut.edu.cn (J.H.); 2 School of Light Industry Science and Engineering, South China University of Technology, Guangzhou 510640, China

**Keywords:** epoxy, radical grafting polymerization, flame retardant, phosphorus-nitrogen synergism

## Abstract

A novel high-efficient flame retardant epoxy grafted poly-acrylic resin modified by phosphorus and nitrogen was successfully synthesized by radical grafting polymerization and solution polymerization simultaneously. The flame retardancy of copolymer resin was investigated using thermogravimetric analysis (TGA), cone calorimetric test (CONE), limiting oxygen index (LOI) and so on. The micro-morphology and chemical composition of char formed after a CONE calorimetric test was analyzed using scanning electron microscopy (SEM) and X-ray photoelectron spectroscopy (XPS), respectively. The Kissinger method was used to evaluate the kinetics of thermal decomposition on copolymer resin. The results showed that the flame retardant property of copolymer resin increased with the increase in phosphorus content. With the increase in nitrogen content, however, the flame retardant property first increased and then decreased. The flame retardant property of the resin was the best and the limiting oxygen index could reach 34.3% when the phosphorus content and nitrogen content of the copolymer resin were 6.45 wt% and 2.33 wt%, respectively. Meanwhile, nitrogen-containing compounds will interact with phosphorus-containing compounds to form P-N intermediates during combustion, which have stronger dehydration and carbonization and could further enhance the flame retardant performance of the resin and generate phosphorus-nitrogen synergistic interactions.

## 1. Introduction

Poly-acrylate resins are commonly used in textile, papermaking and other industries as coatings and adhesives due to their excellent weather resistance, film-forming properties and reputation for lesser environmental harm [1]. However, poly-acrylate resins burn easily. The usual method to address this is to directly blend poly-acrylic resin with flame retardant agents to obtain flame retardant poly-acrylic resin. However, both the flame retardant property and the comprehensive performance of the blended poly-acrylic resin [2,3] are greatly reduced due to the poor compatibility between flame retardant agents and poly-acrylic resin. At present, many researchers are focused on the introduction of flame retardant elements such as halogens, phosphorus and nitrogen into polymer molecular structures to obtain intrinsic flame retardant poly-acrylic resins [4]. Phosphorus (P) and nitrogen (N) elements have demonstrated good ability as flame retardants for polymer materials due to low toxicity, less smoke and high-flame retardant efficiency [5].

During combustion, the phosphorus element or group of the polymer chain could form phosphoric and polyphosphoric acids that could accelerate the carbonization of the polymer substrate to yield an intumescent physical barrier to heat radiation transfer and diffusion of combustible gases. In addition, phosphorus also takes effect as an efficient radical scavenger to impede combustion by the quenching mechanism in gas phase [6]. The nitrogen containing compounds can decompose to nitrogenous nonflammable gases such as nitrogen or ammonia, which will dilute oxygen concentration at the substrate-flame interface, reducing development of flame and increasing the possibility of self-extinguishment [7]. Obviously, flame retardants containing phosphorus and nitrogen can greatly enhance the flame retardancy of poly-acrylic resins. Many studies have reported the preparation of flame retardant acrylic acid resin using phosphorus and nitrogen [8,9,10]. Their results showed that the prepared phosphorus and nitrogen containing poly-acrylate resin has excellent flame retardant performance. However, there are still many problems to be solved in this respect. For instance, the reaction condition is harsh, and the production procedure is very tedious; current research can hardly improve flame retardant monomer conversion rates so that the phosphorus content of copolymers is limited. Additionally, there is not enough research on the mechanism of phosphorus and nitrogen containing flame retardant, i.e., how does the nitrogen additive positively act on the phosphorus additive to display synergism rather than antagonism.

In our previous research, we reported a polymerizable phosphorus-containing monomer—Hydroxyethyl methacrylate phosphate ester (HPN), which can polymerize with acrylic monomers by semi-continuous emulsion polymerization and then obtain a P-containing polyacrylic resin [1]. The research results showed that the prepared P-containing poly-acrylic resin exhibited excellent flame retardancy. However, it can be seen from the polymerization mechanism that the polymerization reaction generates phosphoric acid, which results in a decrease in the water resistance of polymer resin. At the same time, the separation process will be quite complicated due to their close polarity and other properties. Therefore, it is necessary to remove phosphoric acid without affecting the comprehensive properties of HPN monomer. The most ideal method is to introduce phosphoric acid into the polymer molecular chain, which not only reduces the temperature of the condensed phase, but also improves the flame-retardment.

In this paper, bisphenol A epoxy resin (E-51) was introduced into the polymer to achieve the above purpose. There are four main reasons. Firstly, the epoxy group of bisphenol A epoxy resin can react with phosphoric acid to generate epoxy phosphate (EPPA) [11], so as to eliminate phosphoric acid from HPN. Secondly, the Tertiary (secondary) carbon atoms of bisphenol A epoxy resin molecule also have radical polymerization activity, which will lose hydrogen atoms to produce active graft sites under the attack of a free radical, and then free radical graft copolymerization occurs with the double bonds of HPN monomer and acrylic monomer. In this way, phosphoric acid was introduced into the molecular chain of acrylic resin, and the content of phosphorus in the resin is further increased. Thirdly, the excessive epoxy resin can continue the ring-opening condensation reaction with the phosphoric hydroxyl group, thus enabling the graft copolymer to form a network structure through self-crosslinking. Lastly, but also significantly, epoxy resin has excellent characteristics such as good toughness, low shrinkage, strong adhesion to base material and strong generality of formula, which can further improve the comprehensive performance of poly-acrylic resin.

Furthermore, urea, a nitrogen containing monomer, which could form a stable amino structure coordination compound with the phosphorus hydroxyl group from the prepared P-containing poly-acrylate resin system, was introduced to obtain a phosphorus and nitrogen flame retardant modified epoxy acrylic resin (HPN-EPPA-UR-PA) and then further enhanced the comprehensive properties of the poly-arylate resin. The molecular structure, flame-retardant property and thermal property of the prepared resin were characterized by FTIR, SEM, LOI, TG-IR and CONE, etc. The thermal decomposition kinetics of the resin were studied using the Kissinger method. Furthermore, we described the effect of P-N synergistic flame retardation and its interaction mechanism, which may contribute to the further understanding of the mechanism of P-N synergistic flame retardation and the development of new P-N flame retardants in the future.

## 2. Experimental

### 2.1. Materials

Butyl acrylate (BA), Hydroxyethyl methacrylate (HEMA) and Methacrylic acid (MAA) were purchased from Guangzhou Chemical Reagent Factory, China. Phosphorus pentoxide (P_2_O_5_) and urea (UR) were provided by Tianjin Damao Chemical Reagent Factory, China. Bisphenol A epoxy resin (E-51) was supplied by Shanghai Aladdin China Co., Ltd., China. Ammonia (NH_4_OH), Anhydrous ethanol and 2,2’-Azobis (2-methylpropionitrile) (AIBN) were purchased from Shanghai Wokai Chemical Reagent Factory, China. Deionized water is homemade for the laboratory.

### 2.2. Preparation of Phosphorus-Nitrogen Modified Acrylic Resin (HPN-EPPA-UR-PA)

#### 2.2.1. Preparation of HPN Monomer

**Figure 1:**
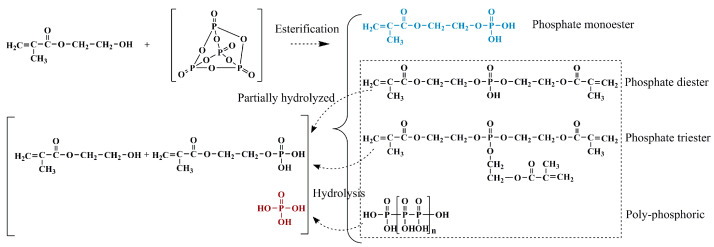
Synthesis route of HPN monomer.

HPN monomer was synthesized according to our previous work [12] with some adjustments. The molar ratio of raw material was changed to HEMA:P_2_O_5_ = 2.6:1. The condensation reaction can proceed between the −OH group of HEMA and the O=P−O group from the P_2_O_5_. The reaction mechanism is as shown in Figure 1.

**Figure 2:**
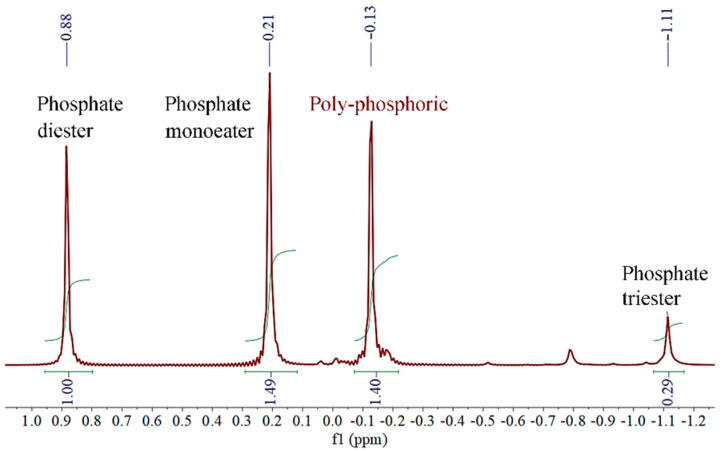
^31^P NMR of HPN monomer.

Due to the fact that the chemical structure of P_2_O_5_ has three O=P−O groups, theoretically, the obtained product might include mono-phosphate ester, di-phosphate ester, tri-phosphate ester and pyrophosphoric acid. The obtained product was analyzed by ^31^P NMR, shown in Figure 2. Data are reported as follows: Phosphate monoester, δ = 0.21 ppm(s); Phosphate diester, δ = 0.88 ppm(s); Phosphate triester, δ = −1.11 ppm(s); Poly-phosphoric, δ = −0.13 ppm(s).

#### 2.2.2. Preparation of Phosphorus Modified Poly-Acrylic Resin

**Figure 3:**
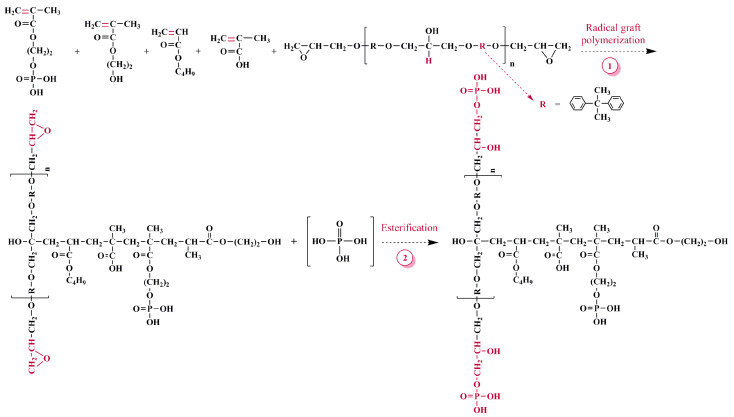
Synthesis route of phosphorus modified poly-acrylic resin.

Then, HPN monomer, E-51 and acrylic monomer were used as raw materials for copolymerization through solution polymerization and graft polymerization simultaneously. All the polymerizations were carried out in a 500 mL four-neck flask equipped with reflux condenser, mechanical stirrer, dropping funnels, and heated in the water bath. First, an appropriate amount of E-51 and AIBN was introduced into the four-neck flask charged with an appropriate amount of Anhydrous ethanol. The temperature was raised to 65 °C and the stirring rate was kept at about 200 rpm. According to the formula, HPN monomer and acrylic monomer (BA and MAA) were weighed, and 1/3 of each was added to the four-mouth flask, which was heated to 75 °C and reacted for 0.5 h. The remaining acrylic monomer mixed with the remaining E-51, HPN monomer and the remaining AIBN dissolved in anhydrous ethanol were added drop by drop into the flask simultaneously, the process lasting 2 h, and then the reaction continued for 3 h. The synthesis route is as depicted in Figure 3.

#### 2.2.3. Preparation of Phosphorus-Nitrogen Modified Poly-Acrylic Resin (HPN-EPPA-UR-PA)

**Figure 4:**
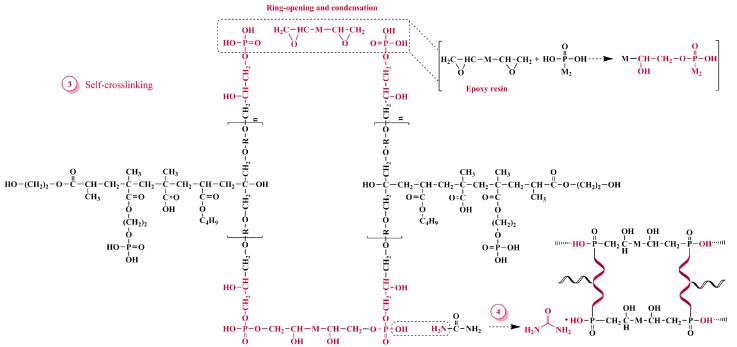
Synthesis route of HPA-EPPA-UR-PA resin.

An appropriate amount of urea was added into prepared phosphorus modified poly-acrylic resin and continued the reaction for 1.5 h and then cooled to room temperature to obtain HPN-EPPA-UR-PA resin. The synthesis route is shown in Figure 4 and its recipe is presented in Table 1.

### 2.3. Characterization

#### 2.3.1. Fourier Transform Infrared Spectroscopy (FTIR)

A small amount of the prepared modified poly-acrylic resin solution was added to the KBr powder, then ground, dried and pressed into a tablet for FTIR test. FTIR spectra analyses of the resulting copolymers was carried out on a FTIR spectrometer (TENSOR27, Bruker, Germany) with the wavenumber range set to 4000~400 cm^−1^.

#### 2.3.2. Thermogravimetric Analysis (TGA)

TGA was carried out on a thermogravimetry analyzer (TG209F1, Netzsch, Germany). The TGA curve of the sample was collected at a temperature range of 30−600 °C under a nitrogen flow of 30 mL/min. The heating rate was 10 °C/min.

#### 2.3.3. Thermogravimetry-Fourier Transform Infrared Spectrometry (TG-IR)

The TG-FTIR was carried out on a thermogravimeter (TG209F1, Netzsch, Germany), which was connected with an infrared spectrometer (Tensor 27, Bruker, Germany). The spectra were collected at a temperature range of 30−800 °C under a nitrogen flow of 30 mL/min. The heating rate was 20 °C/min.

#### 2.3.4. Flame Retardment Tests

The limiting oxygen index (LOI) test was carried out on an oxygen index meter (JF-3, Nanjing Jiangning Analytical Instrument Co., Ltd., Nanjing, China) according to ASTM D 2863−2008 [13] with a sample size of 80 mm × 10 mm × 4 mm.

The cone calorimetric test (CONE) was carried out on a cone calorimeter (6810, Suzhou Vouch Testing Technology Co., Ltd., Suzhou, China) according to ISO 5660-1 [14] with a sample size of 100 mm × 100 mm × 4 mm. One side of the sample was wrapped with aluminum foil and the other side was exposed to a vertical heat flux of 35 kW/m^2^. The heat release and smoke production data were collected.

#### 2.3.5. Scanning Electron Microscopy (SEM)

The surface layer of char yield of copolymer after the CONE test was characterized by SEM. SEM was carried out on a field emission scanning electron microscope (Merlin, Carl Zeiss, Jena, Germany).

#### 2.3.6. X-ray Photoelectron Spectroscopy (XPS)

The surface layer of char yield of copolymer after the CONE test was characterized by XPS. XPS was carried out on an X-ray photoelectron spectrometer (Axis Ultra DLD, Shimadzu, Japan). Al-Kα X-ray (hν = 1486.6 eV) was used as the excitation source and the beam spot size was 700 μm × 300 μm. The accelerating voltage and current were 15 kV and 5 mA, respectively. Data were recorded at the take-off angle of 90°.

## 3. Results and discussion

### 3.1. FTIR Spectra of HPN-EPPA-UR-PA Resin

**Figure 5:**
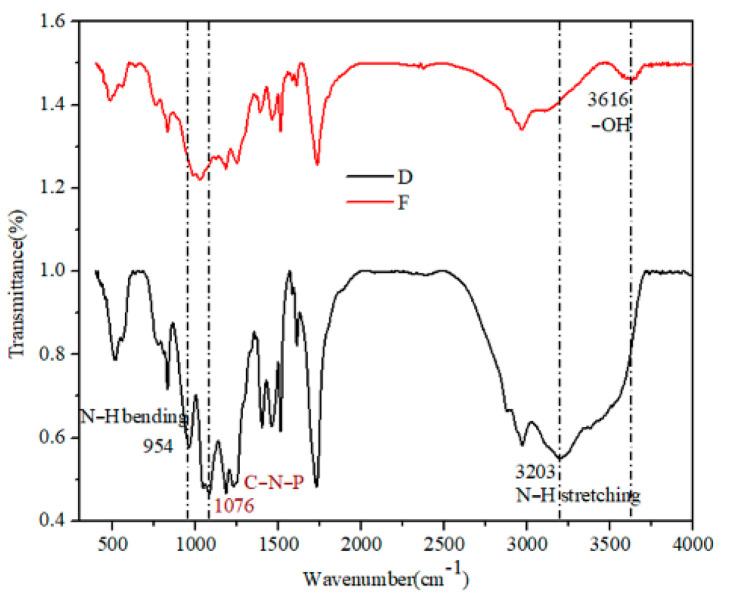
Infrared spectra of HPN-EPPA-UR-PA resin, D: 10 wt% urea; F: 0.

The molecular structure of copolymer resin was analyzed by infrared spectroscopy, and the FTIR spectra are shown in Figure 5.

It can be seen from Figure 5 (sample F) that the absorption peak of hydroxyl (−OH) at 3616 cm^−1^ can be observed, while the absorption peak of sample D at 3616 cm^−1^ was not obvious, indicating the successful reaction between urea and phosphorus hydroxyl. In addition, three new absorption peaks appeared in sample D at 954 cm^−1^, 1076 cm^−1^ and 3203 cm^−1^, corresponding to N−H bending vibration absorption peak, C−N−P [15] and N−H stretching vibration absorption peak, respectively, which further indicated that urea molecules had been successfully incorporated into the molecular structure of the copolymer resin.

### 3.2. Residual Phosphoric Acid Content of HPN-EPPA-UR-PA Resin

**Figure 6:**
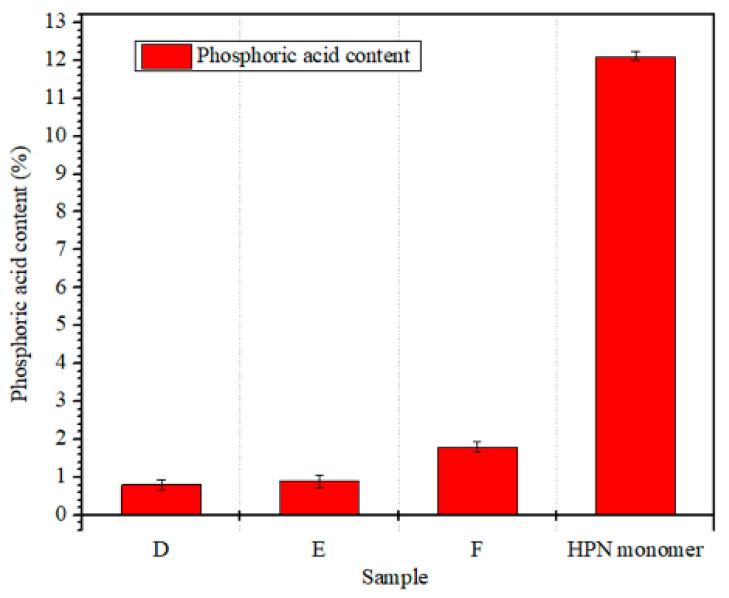
Phosphoric acid content of HPN-EPPA-UR-PA resin, D: 10 wt% urea; E: 5 wt% urea; F: 0.

The residual phosphoric acid content of the copolymer resin system was determined by double indicator titration [15], and the results are shown in Figure 6.

As can be seen from Figure 6, compared with HPN monomer, the phosphoric acid content of copolymer resin was significantly reduced. This indicates that E-51 fully reacts with free phosphoric acid contained in the HPN monomer, and then forms epoxide phosphoric acid (EPPA). In addition, the phosphoric acid content of HPN-EPPA-UR-PA resin will be further reduced after the introduction of urea. When the amount of urea was 5% (formula E), the phosphoric acid content of the copolymer system decreased to 0.9%, and the phosphoric acid content of the copolymer system remained almost unchanged when the amount of urea continued to increase to 10% (formula D).

### 3.3. Thermostability of HPN-EPPA-UR-PA Resin

**Figure 7:**
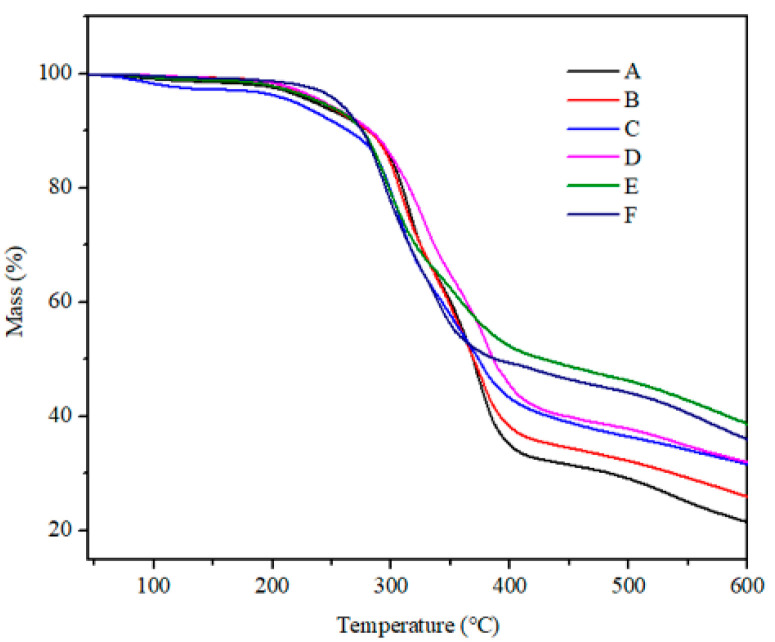
Thermogravimetric curve of HPN-EPPA-UR-PA resin.

**Figure 8:**
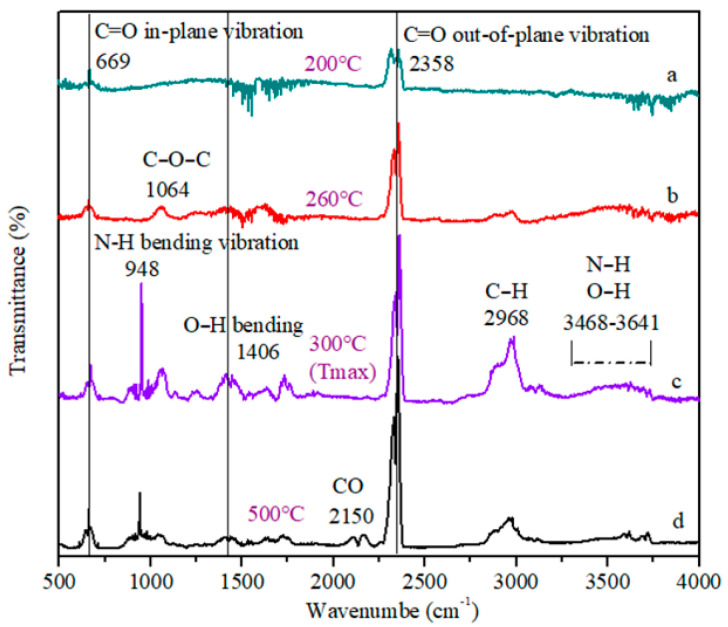
Thermal decomposition gas phase infrared spectrum of HPN-EPPA-UR-PA resin (Formula D) at different temperatures, a: 200 °C; b: 260 °C; c: 300 °C ($T_{max}$); d: 500 °C.

The TGA of HPN-EPPA-UR-PA resin with various phosphorous content was tested and the results are shown in Figure 7 and Table 2. TG-IR curves of HPN-EPPA-UR-PA resin are shown in Figure 8. From Figure 7 and Table 2, we can see that the initial decomposition temperature ($T_{i}$) of HPN-EPPA-UR-PA resins was about 240 °C, and the maximum decomposition rate temperature ($T_{max}$) gradually decreased with an increase in the amount of HPN monomer (i.e., increase in phosphorus content). Phosphorus-containing compounds break down into phosphoric acid at lower temperatures, which catalyzes the dehydration of the resin to form a char layer. With the increase in the phosphorus content of copolymer resin, the catalytic dehydration rate will be accelerated, which will accelerate the resin mass loss rate, and thus reduce the maximum decomposition rate temperature ($T_{max}$) [16]. Additionally, the surface resin gradually dehydrates and carbonizes as the thermal decomposition reaction goes on. After forming a considerable structured char layer, its heat insulation and quality insulation effect will be significant, and greatly reduces the decomposition rate of resin and mass loss. Meanwhile, the char forming rate and its amount of the resin increased with the increase in the phosphorus content of HPN-EPPA-UR-PA resin, resulting in the increase in the residue rate of the copolymer resin. The increase in residue rate also leads to the increase in the LOI value of the resin, and there is a good consistency between them.

As is shown in Figure 8, the composition of gas evolved was confirmed by TG-IR analysis during the pyrolysis of HPN-EPPA-UR-PA resin, with the characteristic peaks of C=O at 669 cm^−1^ and 2358 cm^−1^, and N−H at 948 cm^−1^ and 3468 cm^−1^. The results showed that the copolymer resin can release CO_2_, NH_3_ and other noncombustible gases during combustion, which play the role of gas phase flame retardant.

Compared with formulas D, E and F, we can see that the residue of the resin first increased and then decreased with the increase in nitrogen content. The copolymer resin will decompose and release non-flammable gases such as NH_3_ with the increase in nitrogen content, thus diluting the oxygen concentration in the gas phase and taking away some of the heat, then leading to the increase in residue rate. However, if the nitrogen content continues to increase, the gas release rate will accelerate, which may destroy the char layer formed on the surface. In serious cases, the inner layer of resin will be exposed, and the heat will quickly enter into the resin, intensifying the decomposition of the resin system and resulting in the decrease in residue rate.

### 3.4. Flame Retardancy of HPN-EPPA-UR-PA Resin

**Figure 9:**
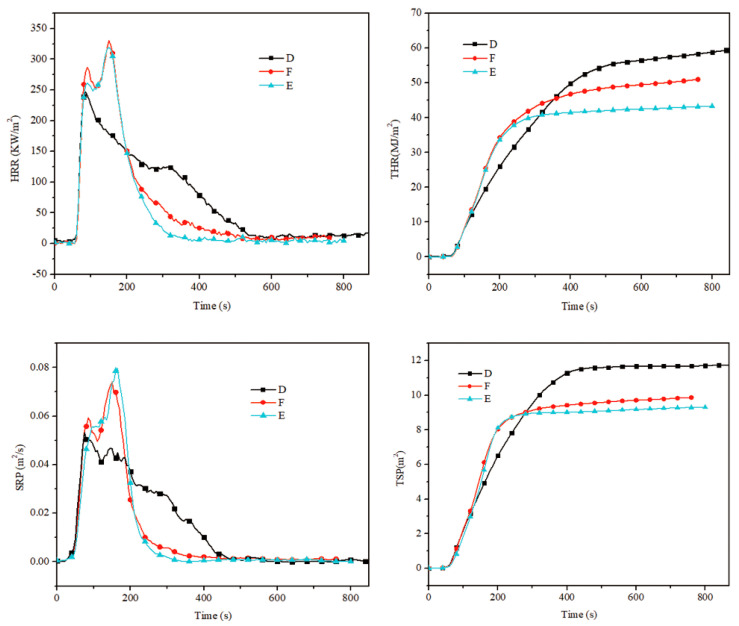
The CONE curve of HPN-EPPA-UR-PA resin, D: 10 wt% urea; E: 5 wt% urea; F: 0.

The flame-retardant property of HPN-EPPA-UR-PA resin was tested by a cone calorimeter. The heat release rate (HRR), total heat release (THR), smoke production rate (SPR) and total smoke production (TSP) curves of copolymer resin are shown in Figure 9 and the corresponding data is listed in Table 3.

The heat release rate (HRR) and smoke production rate (SPR) of formula E tend to zero after burning for 300 s, while formulas D and F tend to zero after burning for 500 s. Meanwhile, the total heat release rate (THR) and total smoke production (TSP) of formula E are both lower than those of formulas D and E. In addition, the heat release rate and smoke release rate of formula D are low at the beginning of the combustion; this is mainly due to the high content of urea (nitrogen). However, in the middle and later periods of the combustion, the heat release rate and smoke release rate are highest, which was caused by the destruction of the surface residue structure; hence, it cannot perform well as a flame retardant. These results are consistent with the TGA analysis results. As can be seen from Table 3, the average heat release rate, total heat release and average effective heat of combustion are the lowest in formula E. Compared with formula D, those rates were reduced by 18.23%, 28.22% and 51.10%, respectively. This further illustrates that urea has great influence on the flame retardant property of the resin. In other words, at a certain amount of nitrogen, the resin can show the synergistic flame retardant effect of phosphorus and nitrogen, and nitrogen compounds can promote the dehydrating carbonization and expansion flame retardant effect of phosphorous compounds, which would endow the resin with excellent flame retardant performance.

### 3.5. Morphology and Chemical Composition Analysis of the Char Residue

**Figure 10:**
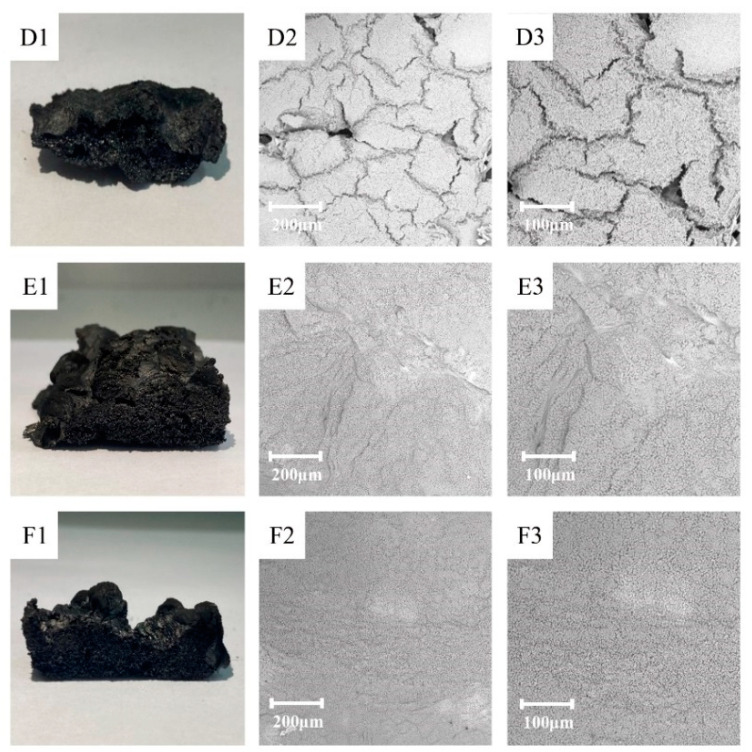
(**D1**,**E1**,**F1**) show the morphology of char layers; (**D2**,**E2**,**F2**) were taken at ×300 magnification; (**D3**,**E3**,**F3)** were taken at ×500 magnification.

Figure 10 shows the SEM results of the char layers of HPN-EPPA-UR-PA resin after CONE analysis. There was obvious difference in the morphology of the char layers of formulas D, E and F. After prolonged burning at high temperature, the surface char layer of formula D was full of cracks, thus exposing the inner layer structure, which led to heat entering the resin and exacerbating the thermal degradation of the resin. The surface char layers of formulas E and F were compact and intact. Among them, formula E has an obvious expansion action, indicating that it can have a better heat insulation effect, so it has the best flame-retardant performance. The expansion action may be due to the gas produced by the thermal decomposition of urea and the foaming action of nitrogenous compounds. SEM results further proved that the addition of urea (nitrogen) had a significant effect on the flame retardant property of HPN-EPPA -UR-PA resin.

**Figure 11:**
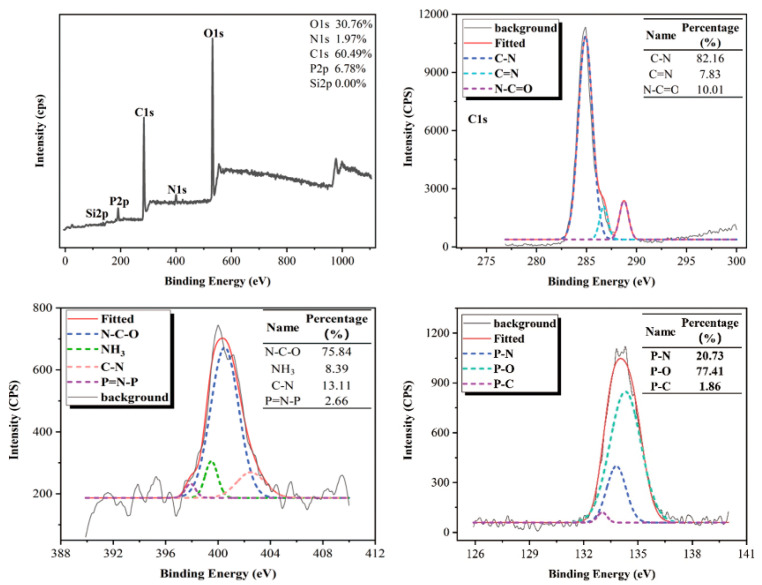
XPS spectra of the char layer of HPN-EPPA -UR-PA resin (Formula E).

In order to further investigate the influence of nitrogen on the chemical structure of the products in the catalytic carbonization reactions, XPS was used to analyze the char layer of HPN-EPPA -UR-PA resin. The results are shown in Figure 11. C spectrum existed in forms of C−N (285.8 eV), C=N (287.0 eV) and N−C=O (288.2 eV). C−N accounted for the vast majority. N spectrum existed in forms of N−C−O (399.6 eV), NH_3_ (399.8 eV) and C−N (402.4 eV), and P spectrum existed in forms of P−O (135.1 eV), P−C (133.0 eV) and P−N (132.9 eV) [17]. This result indicated that the nitrogen element was involved in the carbonization reaction. During combustion, nitrogen-containing compounds interact with phosphorus-containing compounds to form P-N intermediate, which is a stronger dehydrating agent, accelerating the dehydration and carbonization rate of the resin, thus further improving its flame retardant performance.

### 3.6. Kinetics of Thermal Degradation of HPN-EPPA -UR-PA Resin

Activation energy of thermal decomposition is one of the important kinetic parameters of thermal degradation reaction, and its value changes with the progress of thermal decomposition [16,18]. In order to further understand the process of oxidative thermal decomposition of HPN-EPPA-UR-PA resin and the effect of nitrogen content on the flame retardant property of the resin, the thermal decomposition kinetics of the resin were explored by using the Kissinger method. The higher the decomposition activation energy, the better the thermal stability. The differential method for the determination of kinetic parameters is an important method for the determination of decomposition activation energy. It is calculated using the change in thermogravimetric data caused by the variation in temperature rate. The formula for decomposition activation energy calculated by the Kissinger method is as follows:

(1) $$\ln\frac{\beta }{T^{2}}=\left\{\ln\frac{AR}{E}+\ln\left[n{\left(1-\alpha \right)}^{n-1}\right]\right\}-\frac{E}{RT}$$

where *T* is the thermodynamic temperature, *α* is a conversion at *T*, n is the reaction order, and *A* is the pre-exponential factor. According to the Kissinger approximation, $f^{\prime }\left(\alpha_{max}\right)=n{\left(1-\alpha_{max}\right)}^{n-1}≅1$ , therefore:

(2) $$\ln\frac{\beta }{T_{max}{}^{2}}=\left\{\ln\frac{AR}{E}\right\}-\frac{E}{RT_{max}}$$

Thus, a set of thermogravimetric curves was determined at different temperature-rates to obtain a set of corresponding $T_{max}$. The activation energy *E* can be determined from a plot of $\ln\left(T_{max}^{2}/\beta \right)$ against $\left(1/T_{max}\right)$. The activation energy is then obtained from E=S×R (*S* is the slope of the line and *R* is the gas constant).

#### 3.6.1. Decomposition Activation Energy of Resin with Different Phosphorus and Nitrogen Content

**Figure 12:**
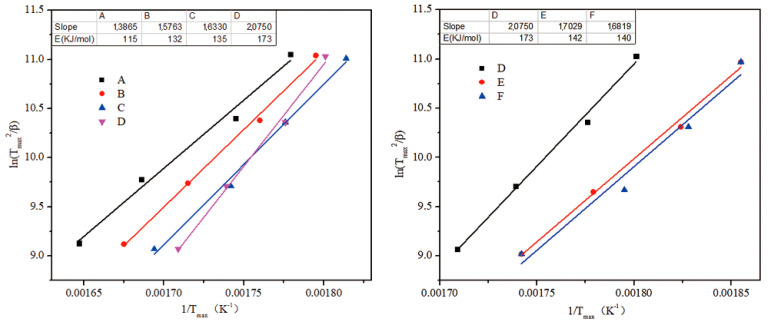
Decomposition activation energy of HPN-EPPA-UR-PA resin with different phosphorus and nitrogen content.

A set of thermogravimetric curves was obtained with different temperature-rates (5 °C/min, 10 °C/min, 20 °C/min, 40 °C/min). The thermal decomposition activation energy of different resin formulas under the maximum decomposition rate temperature ($T_{max}$) was calculated by using the Kissinger method. The results are shown in Figure 12.

As shown in Figure 12, at the maximum decomposition rate temperature ($T_{max}$), the decomposition activation energy of copolymer resin gradually increased with the increase in the amount of HPN monomer (phosphorus content). At the same phosphorus content, its decomposition activation energy also increased with the increase in urea content (nitrogen content). When the amounts of HPN monomer and urea were 50 wt% and 10 wt% respectively, the decomposition activation energy of copolymer resin was the highest (173 KJ/mol). However, thermogravimetric analysis showed that the thermal stability of formula D was not the best, and it was even lower than formula F. This is because, compared with formulas E and F, the urea content in formula D is so high that its activation energy is high at the initial stage of thermal decomposition, which may be the activation energy of the extra portion of urea. With the process of thermal decomposition, the release rate of non-combustible gases such as NH_3_ was accelerated, which may destroy the char layer generated on the surface. This weakened its heat insulation effect, which exacerbated the thermal decomposition of the inner resin and resulted in a lower residue rate than formulas E and F. This indicates that the thermal stability of copolymer resin is not only related to its own properties (decomposition activation energy), but also related to the structural changes in the char layer generated on the surface during the thermal decomposition process, i.e., the integrity and compactness of chars on the surface after combustion were the key to the flame retardant performance of HPN-EPPA-UR-PA resin.

#### 3.6.2. Decomposition Activation Energy of Copolymer Resin at Different Residue Rate

**Figure 13:**
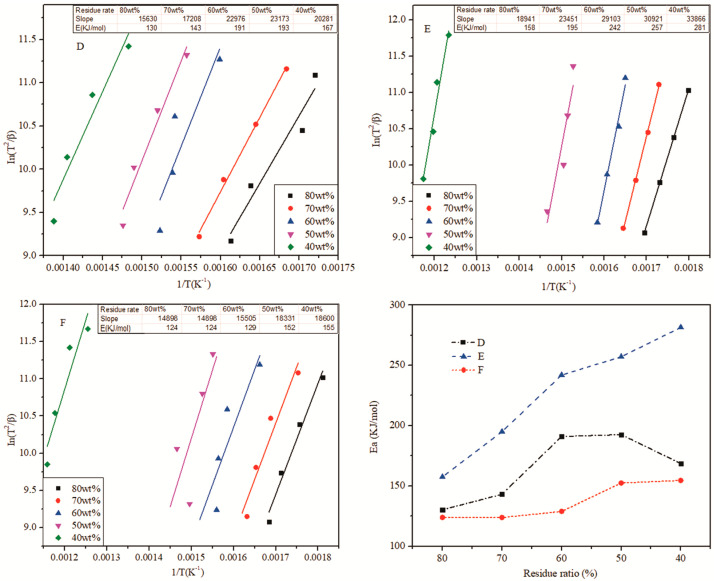
Decomposition activation energy of HPN-EPPA-UR-PA resin and its variation trend under different residue rates, D: 10 wt% urea; E: 5 wt% urea; F: 0.

Figure 13 shows the correspondence and variation tendency of the residue rates of formulas D, E and F and their decomposition activation energy. The decomposition activation energy was calculated at 80 wt%, 70 wt%, 60 wt%, 50 wt% and 40 wt%, respectively, so as to obtain the change in the decomposition activation energy of HPN-EPPA-UR-PA resin within the thermal decomposition process [19].

The results showed that at the initial stage of thermal decomposition, the decomposition activation energy of formula D increased with the progress of thermal decomposition when the carbon residue rate was higher than 60 wt%. However, when the residue rate was lower than 50 wt%, its decomposition activation energy decreased sharply. This result is consistent with the TGA data, and was caused by the destruction of the surface char layer structure in the later stage of thermal decomposition, followed by the heat entering into the resin and intensifying its decomposition. The activation energy of formula F increases gradually with the progress of thermal decomposition. When the residue rate is less than 50 wt%, the activation energy increases slowly. This is because there is no addition of urea in formula F, and the increase in activation energy is only due to the catalytic carbonization of phosphorous substances. In the later stage of thermal decomposition, the structure of the char layer was complete, and the heat insulation effect was significant, thus the activation energy did not increase significantly. The activation energy of formula E strictly increased with the progress of thermal decomposition, which may be caused by the synergistic effect of phosphorus and nitrogen. Phosphorus-containing compounds form an expansive char layer at high temperature that acts as a protective layer for heat insulation and oxygen resistance, while nitrogen-containing compounds also act as foaming agents and char enhancers, which will further increase the flame-retardant effect of phosphorus-containing compounds. The above results indicate that higher urea content (nitrogen content) is not necessarily better. Within a certain range, it may show the synergistic flame retardation effect of phosphorus and nitrogen. Beyond this range, however, there is no significant synergy.

## 4. Conclusions

The HPN-EPPA-UR-PA resin with excellent flame retardant property was successfully prepared by radical grafting polymerization and solution polymerization simultaneously. The results of double indicator titration showed that most of the phosphoric acid in HPN monomer could react successfully with E-51 of the resin system. The results of TGA, SEM and CONE showed that the flame retardant property of copolymer resin first increased and then decreased with the increase in nitrogen content at the same phosphorus content. TG-IR results showed that non-flammable gases such as CO_2_, NH_3_ and water vapor will be produced when the resin is decomposed by heat. The results of thermal decomposition kinetics showed that when the urea content in the copolymer resin was low, it could produce a phosphorus and nitrogen synergistic flame retardant effect. However, there was no obvious synergistic effect when the urea content was excessive.

## Figures and Tables

**Table 1:** Recipe of HPA-EPPA-UR-PA resin.

Formula	HPN	E-51	BA	MAA	UR	P Content/wt%	N Content/wt%
A	20	25	40	5	10	2.58	4.66
B	30	25	30	5	10	3.87	4.66
C	40	25	20	5	10	5.16	4.66
D	50	25	10	5	10	6.45	4.66
E	50	25	15	5	5	6.45	2.33
F	50	25	20	5	0	6.45	0.00

**Table 2:** Decomposition temperature and carbon residue of HPN-EPPA-UR-PA resin.

Formula	$T_{i}/{}^{\circ}C$	$T_{max}/{}^{\circ}C$	Residue Rate (600 °C)/%	LOI/%
A	235.1	303.6	21.5	22.4
B	239.5	295.7	25.9	26.9
C	236.4	290.7	31.6	28.4
D	241.3	290.4	32.0	30.1
E	239.4	272.8	38.6	34.3
F	254.7	275.2	35.9	31.5

Note: $T_{i}$ represents the initial decomposition temperature (the temperature at weight loss of 5 wt%), and $T_{max}$ represents the maximum decomposition rate temperature.

**Table 3:** Data of cone calorimeter of HPN-EPPA-UR-PA resin.

Formula	D	E	F
AV-HRR/KW/m^2^	141.06	115.35	130.94
THR/MJ/m^2^	60.43	43.38	51.07
AV-EHC/MJ/kg	18.26	8.93	10.62
TRP/m^2^	11.74	9.29	9.87
Weight loss rate/g/s	0.068	0.062	0.065
Total oxygen consumption/g	33.70	24.18	29.04
CO_2_ production rate/kg/kg	1.29	0.72	0.88

Note: AV-HRR refers to the average heat release rate; AV-EHC refers to the average effective heat of combustion.
